# Glycopeptide-Based Antibody Detection in Multiple Sclerosis by Surface Plasmon Resonance

**DOI:** 10.3390/s120505596

**Published:** 2012-05-02

**Authors:** Feliciana Real-Fernández, Irene Passalacqua, Elisa Peroni, Mario Chelli, Francesco Lolli, Anna Maria Papini, Paolo Rovero

**Affiliations:** 1 Laboratory of Peptide & Protein Chemistry & Biology, Polo Scientifico e Tecnologico, University of Florence; I-50019 Sesto Fiorentino (FI), Italy; E-Mails: real@toscanabiomarkers.com (F.R.-F.); irene.passalacqua@gmail.com (I.P.); mario.chelli@unifi.it (M.C.); lolli@unifi.it (F.L.); annamaria.papini@unifi.it (A.M.P.); 2 Department of Chemistry “Ugo Schiff” and CNR ICCOM, Via della Lastruccia 3/13, University of Florence, I-50019 Sesto Fiorentino (FI), Italy; 3 Department of Pharmaceutical Sciences, Via Ugo Schiff 6, University of Florence, I-50019 Sesto Fiorentino (FI), Italy; 4 Laboratoire SOSCO EA 4505, University of Cergy-Pontoise, 5 mail Gay-Lussac, Neuville sur Oise, Cergy-Pontoise cedex 95031, France; E-Mail: elisa.peroni@u-cergy.fr; 5 Department of Neurological Sciences & Azienda Ospedaliera Universitaria di Careggi, Viale Morgagni 34, University of Florence, I-50134 Firenze (FI), Italy

**Keywords:** multiple sclerosis, surface plasmon resonance, serodiagnosis, biacore, immunoassay, glycopeptide CSF114(Glc)

## Abstract

In multiple sclerosis (MS) the gold standard for the diagnosis and prognosis is, up to now, the use of magnetic resonance imaging markers. No alternative simpler assays proven of use, except for cerebrospinal fluid analysis, have been provided in MS diagnosis. Therefore, there is a need to develop non-invasive, sensitive, simple new techniques for the clinical routine. Herein we present the evaluation of the feasibility of a glycopeptide-based biosensor to detect MS specific antibodies in sera using the surface plasmon resonance technology. The previously described glycopeptide antigen CSF114(Glc) has been immobilized on a gold sensor chip and the method has been optimized for real-time specific autoantibody detection directly in sera. A population of 60 healthy blood donors and 61 multiple sclerosis patients has been screened. The receiver operating characteristic (ROC)-based analysis has established the optimal diagnostic cut-off value for the method obtaining a sensitivity of 36% and a specificity of 95%. Sample sera have been also screened with a previously validated ELISA.

## 1. Introduction

Multiple sclerosis (MS) is a chronic inflammatory disease involving different areas of the central nervous system [1]. In the setting of many distinctive neuropathological features present in the disorder, which suggest different pathophysiological mechanisms leading to the formation of myelin lesions [2,3], the disease pathogenesis is most probably linked to an autoimmune mechanism against myelin antigens in the central nervous system [4].

Magnetic resonance imaging (MRI) is up to now the most reliable technique, not only for MS diagnosis, but also for prognosis [5,6]. No alternative simpler assays proven of use, aside from cerebrospinal fluid analysis (CSF), are used in MS diagnosis [7,8]. However, it is evident that MRI and CSF cannot be easily considered as routine and standardized tests. Moreover, the symptoms are not entirely correlated to the clinical setting, and even a targeted MRI may fail.

The autoantigens leading to the autoimmune aggression in MS are still unclear, due mostly to their scarcity and difficult analysis. Growing evidence indicates that post-translational modifications, either native or aberrant, may play a fundamental role in specific autoantibody recognition in autoimmune diseases [9]. MS patients often produce multiple types of autoantibodies which can be present in tissues and biological fluids. Antibodies can then be identified as biomarkers and used to set up diagnostic/prognostic tools [10,11]. Moreover, when antibody fluctuate with disease exacerbations or remissions, they can usefully be measured to monitor the efficacy of a therapeutic treatment [12,13]. Usually, in autoimmune diseases, the autoantibody response begins against one or few autoantigens, then extends in individual responses to numerous others in a process called epitope spreading, and it is to be tested if this process may predict specific clinical manifestations, disease severity and progression. From another point of view, the basic physiological and pathological reactions in the autoimmune responses depend on the discrete antibody binding pattern, the avidity, the molecular specificity and the antibody titres. Affinity/avidity of autoantibodies has not been sufficiently studied and challenged in the diagnostic hypothesis testing. With all these considerations in mind, it is evident that if a single test could definitely enter in the clinical routine, it should reflect such polyclonal responses.

In our laboratories, it was demonstrated that the synthetic glucosylated myelin oligodendrocyte glycoprotein fragment (Asn^31^(Glc)hMOG(30–50)) was able to detect autoantibodies in MS patients' sera by enzyme-linked immunosorbent assay [14]. The ability of the glucosylated sequence to detect autoantibodies in multiple sclerosis patients' sera was correlated to the *N*-linked glucosyl moiety [15]. Hence, the recognition properties of the molecule were optimized through the design and screening of focused libraries of glycopeptides by a “Chemical Reverse Approach”, which led to the development of a specific antigenic probe, termed CSF114(Glc). An immunoenzymatic assay based on this synthetic glycopeptide was shown to identify autoantibodies in patients' sera as biomarkers of multiple sclerosis [16,17]. The glycopeptide is characterized by a β-turn structure bearing as minimal epitope a β-d-glucopyranosyl moiety linked to an Asn residue on the tip of the turn [18,19], possibly reproducing an aberrant *N*-glucosylation of myelin proteins fundamental for autoantibody recognition [20,21].

The enzyme-linked immunosorbent assay (ELISA) is a simple and relatively inexpensive technique offering advantages such as simultaneous analyses of a large number of samples. However, non-specific ‘matrix effects’, or failure or heightened detection of low-affinity, and background antibodies are some accepted disadvantages of the assay [22,23]. Consequently, there is a need for sensitive and more consistent techniques to follow up disease activity. These new assays should retain all the advantages of ELISA, like the possibility of a good standardization, a low cost, and the possibility to identify subclasses of antibody response.

Biosensor technology based on surface plasmon resonance (SPR) has become increasingly popular for monitoring binding interactions [24–26]. SPR technique is extremely interesting in biological and clinical assays because it has the potential to directly visualize biomolecular interactions in real-time [27,28]. In this optical method the ligand is covalently linked on the biosensor surface and the specific analyte is perfused onto this surface, thus allowing quick ligand recognition and binding [29]. Binding on the biosensor surface is expressed graphically in sensorgrams that depict accumulation of mass over time providing instantaneous data. Other advantages of SPR technology include the ability to reuse sensor chips for serial analysis and eradicate the need for labelled reagents providing a rapid one-step analytical methodology. Optical label-free devices have been infrequently used for detection of disease specific antibodies directly in patients' sera. Although published results in this field are fundamentally based on spiked serum samples or on a sandwich assay set-up [30–33] analyses can also be performed directly on crude serum samples [34–36].

Herein we present the evaluation of the feasibility of a glycopeptide-based biosensor to detect MS specific antibodies in sera by a SPR assay. The glycopeptide CSF114(Glc) has been immobilized on a gold sensor chip and used for the screening of healthy blood donors' and MS patients' serum samples. SPR-assay data are compared with the previously validated ELISA method.

## 2. Experimental Section

### 2.1. Reagents

Glycopeptide antigen CSF114(Glc) was prepared by microwave-assisted solid phase peptide synthesis. The glycopeptide was purified to homogeneity by solid phase extraction and reverse phase high-pressure liquid chromatography (HPLC), and further characterized by mass spectrometry and analytical HPLC as described elsewhere [37].

Sensor chip CM5 and the running buffer HBS-EP+ 10× (0.1 M HEPES, 1.5 M NaCl, 30 mM EDTA and 0.5% v/v Surfactant P20; yielded pH 7.4 when diluted) were purchased from Biacore AB (GE Healthcare, Uppsala, Sweden). The amine coupling reagents *N*-hydroxysuccinimide (NHS), 1-ethyl-3-(3-dimethylaminopropyl) carbodiimide hydrochloride (EDC), and 1 M ethanolamine hydrochloride-NaOH pH 8.5 were provided by Biacore AB (GE Healthcare). Sodium acetate was purchased from Carlo Erba (Milano, Italy). Sodium hydroxide was provided by Honeywell-Riedel deHaen (Seelze, Germany). All analyses were performed in a Biacore T100 instrument (GE Healthcare). All experiments were made at 25 °C using HBS-EP+ as running buffer.

### 2.2. Serum Collection

One hundred and twenty one human serum samples were obtained for diagnostic purposes from patients and healthy blood donors who had given their informed consent. Each serum sample was centrifuged and supernatant aliquoted and stored at −20 °C until use. Patients' sera were obtained from a group of 61 relapsing—remitting MS (RR-MS) patients after a diagnostic lumbar puncture. Cerebrospinal fluid analysis and MRI examinations were performed for diagnostic purposes.

### 2.3. Surface Plasmon Resonance (SPR)

A stock solution of glycopeptide CSF114(Glc) was prepared in pure water (1 μg/μL) and stored at +4 °C. Immediately prior to immobilization procedure, peptide stock solution was diluted to a concentration of 10 μg/mL in 0.1 mM sodium acetate pH 5.5. Standard amine coupling procedure was employed for glycopeptide immobilization, essentially according to the Biacore procedures. The appropriate flow cell of the sensor chip surface was activated by injecting an EDC/NHS (50:50) mixture at a flow rate 10 μL/min during 420 s. CSF114(Glc) was injected at 10 μL/min using the *aim of immobilization* procedure to give a final immobilization level of 800 ± 100 Resonance Units (RU). Unreacted groups on sensor chip surface were blocked by injecting 60 s-pulses of 1 M ethanolamine at pH 8.5 until complete deactivation. Reference channel was activated and subsequently blocked with ethanolamine.

All analyses were performed in triplicate at a flow rate of 30 μL/min. Human serum samples were diluted 1:100 and/or 1:50 in running buffer. Samples were injected, at different injection times, in both active and control channels followed by 60 s of buffer injection to allow dissociation. Interaction of samples with sensor chip flow cells were monitored as separate sensorgrams and measurements were taken 15 s after the end of each injection. The antibody responses were measured in RU units as a signal difference between active channel and reference channel. After each measurement, surface was regenerated injecting two pulses of a solution 100 mM NaOH during 60 s.

### 2.4. Enzyme Linked Immunosorbent Assay (ELISA)

The two panels of serum samples were tested in ELISA to check the presence of specific antibodies. Ninety six well activated polystyrene ELISA plates (NUNC Maxisorb; Sigma-Aldrich, Milan, Italy) were coated with 1 μg per 100 μL of glycopeptide CSF114(Glc) per well in pure carbonate buffer 0.05 M (pH 9.6) and incubated at +4 °C overnight. After three washes with saline solution containing 0.05% Tween 20, non-specific binding sites were blocked with fetal calf serum (FCS), 10% in saline Tween 20 (100 μL per well) at room temperature for 60 min. Sera diluted from 1:100 to 1:10,000 were applied at +4 °C overnight in saline solution/Tween 20/10% FCS. After three washes, 100 μL of alkaline phosphatase conjugated antihuman IgM (diluted 1:200 in saline/Tween 20/FCS) or IgG (diluted 1:8,000 in saline/Tween 20/FCS; Sigma-Aldrich) were added to each well. After 3 h incubation at room temperature and three washes, 100 μL of 1 mg·mL^−1^ *p*-nitrophenyl phosphate (Sigma-Aldrich) in 10% diethanolamine substrate solution (pH 9.8) was added. After 30 min, the reaction was stopped with 1 M NaOH (50 μL), and the absorbance was read in a multichannel ELISA reader (Tecan Sunrise, Männedorf, Switzerland) at 405 nm. ELISA plates, coating conditions, reagent dilutions, buffers, and incubation times were preliminary tested [16]. Antibody levels are expressed as absorbance in arbitrary units at 405 nm (sample dilution 1:100).

## 3. Results and Discussion

### 3.1. Biosensor Optimization

To determine MS-specific antibody reactivity against the CSF114(Glc)-based biosensor in a Biacore T100 instrument 60 healthy blood donors' (BD) and 61 MS patients' sample sera were tested. For this purpose glycopeptide CSF114(Glc) was immobilized on the sensor chip surface following the amino coupling chemistry. Reproducible coupling conditions were initially optimized using the *pH scouting* procedure, as described in the instrument protocol, and 0.1 mM sodium acetate pH 5.5 was selected as immobilization buffer.

To establish a reproducible method for autoantibody detection, diluted serum samples were injected over the immobilized glycopeptide at different contact times (60, 120, 180 and 240 s). A 240 s injection was found to be optimum for increasing signal differences between positive and negative samples maintaining low signals in the reference channel. After each sample injection, surface was regenerated with two 60 s pulses of a solution 100 mM NaOH allowing the complete removal of specifically and unspecifically attached material from the surface. Following this protocol all further experiments were performed not over and above 100 measurements per channel.

Biosensor was used for the screening of high positive control sera at dilution 1:100 and 1:50. The analytical variability of the assay was checked repeating the same test (two sera, 15 runs each) or in different experiments (two sera, 12 runs performed once a week). The within-assay and between-assay coefficients of variation (SE/mean) were below 10% for sample dilution 1:100 and below 5% for dilution 1:50. Further serological analyses were performed at sample dilution 1:50, which presented lower signal average. One positive and one negative sample were used as controls each 15 measurements, verifying the stability of the probe upon a large number of cycles. For each measurement a sample volume of 150 μL was employed, thus small amount of 3 μL of patient serum was required for each assay.

### 3.2. Label-Free Serodiagnosis of Multiple Sclerosis

**Figure 1.**
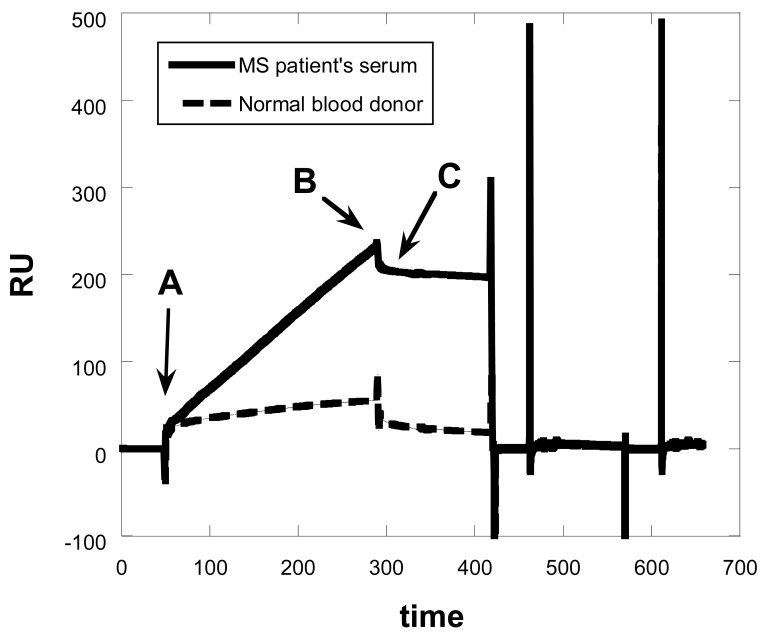
Sensorgram obtained for binding of a MS positive sample and a healthy blood donor sample to the CSF114(Glc)-modified sensor surface. A corresponds to the diluted serum start injection point, which flow through the sensor chip during 240 s. B corresponds to the serum end injection point followed by a buffer wash. C corresponds to the evaluation point 15 s after point B.

Specific antibodies were detectable in some patients' sera. A typical sensorgram obtained when both healthy control and MS patient' sera were injected over the glycopeptide CSF114(Glc) is illustrated in Figure 1. Sensorgram show low association and very weak dissociation. All sample sera presented a similar sensorgram profile.

**Figure 2.**
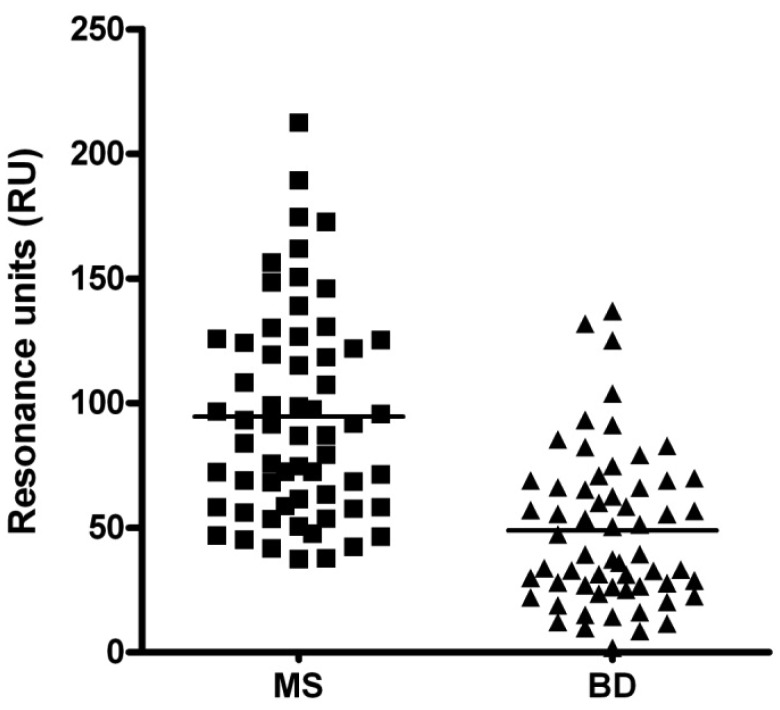
Column scatter and mean values of anti-CSF114(Glc) antibodies (dilution 1:50) in Multiple Sclerosis (MS, n = 61) and blood donors (BD, n = 60). The lines indicate the mean value of each group.

The column scatter of the data reported in Figure 2 summarizes all serological results obtained in the Biacore assays at sample dilution 1:50. The differences between the MS and BD mean values were significant (94.6 *vs.* 48.9 RU respectively), observing the higher values in MS subjects.

**Figure 3.**
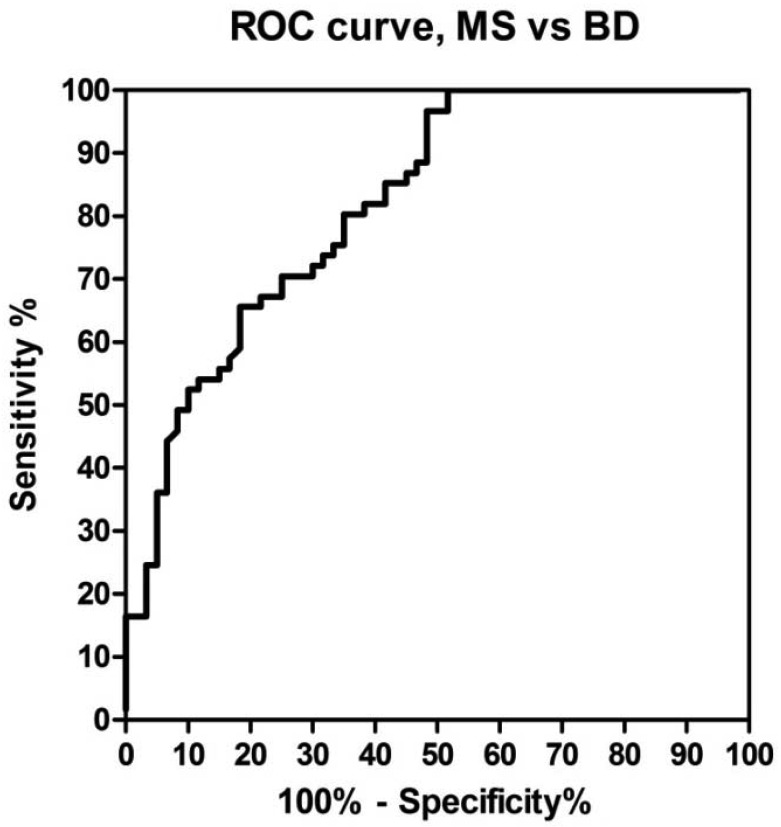
ROC curve analysis of antibodies to CSF114(Glc) in MS *vs.* BD determined by SPR. The area under the curve is 0.82 (p < 0.0001). Cut-off was set at 105 RU with a sensitivity of 36%, a specificity of 95%, and a positive likelihood ratio of 7.21.

A receiver operating characteristic (ROC)-based analysis was employed comparing different cut-off values as sensitivity, specificity and likelihood ratios [38]. ROC curve for anti-CSF114(Glc) activity was constructed based on 61 cases with multiple sclerosis *vs.* 60 controls (Figure 3).

### 3.3. Biacore *vs.* ELISA

The panel of MS and BD sera was also tested against the synthetic peptide CSF114(Glc) following the previously validated ELISA procedure [16]. Both protocols have been evaluated and results compared.

Regarding the coating procedure, immobilization cost in terms of time (2 h aprox) and ligand quantities (1 μg for 100 measurements aprox) in SPR differs clearly from ELISA (coating overnight, 1 μg for each measurement). Moreover, one analysis in SPR can be performed in 10 min compared to at least one day in ELISA. On the other hand, ELISA offers the possibility to perform a large number of measurements simultaneously whereas the Biacore assay cannot analyse more than one sample contemporaneously. Methodologically speaking, SPR protocol appears in any case more convenient than ELISA.

Data obtained in ELISA were further compared with surface plasmon resonance results. Antibody responses were found to be significantly higher in MS than in healthy subjects both in SPR and ELISA. Specificity was similar for both methods, and sensitivity was increased from 26% for ELISA [16] to 36% for the SPR methodology.

Although ELISA exhibited overall results similar to those obtained from the SPR system, in some case only one of the assays was able to recognize specific antibodies. The SPR-based biosensor scored as positives nine MS patients (15%) further screened as negative in ELISA (Table 1, entry 4). Furthermore, the SPR biosensor was able to differenciate as positives seven cases in which ELISA recognized only IgG type antibodies (Table 1, entry 2), and two positive cases of IgM positive in ELISA (Table 1, entry 3). On the other hand, the SPR-biosensor did not detect specific antibody responses whereas the ELISA recognized ten (16%) positive cases to IgM and/or IgG (Table 1, entries 5–7). False positive cases (5%) scored in SPR-assay were similar to those observed in ELISA.

Briefly, immunoassays employed are able to detect glycopeptide CSF114(Glc)-antibody interactions presenting similar sensibility, but increasing sensitivity and recognizing slightly different patient populations, due to the ability of the SPR assay to detect low-affinity antibodies. Besides the two parameters sensitivity and specificity, also affinity can be considered as a parameter since the two methods differ in their underlying principle.

## 4. Conclusions

Herein we have developed a label-free method for the screening of disease-specific antibodies which would offer a protocol with good sensitivity and high specificity obtaining a response within a few minutes. SPR protocol employed only small quantities of glycopeptide antigen CSF114(Glc) and blood serum saving method-cost despite high price of the equipment. Moreover, the sensor surface can be regenerated and reused for more than 100 measurements. In contrast to ELISA, in which information is provided by the use of one specific secondary antibody, the SPR assay yield data directly as the antibody binding to the glycopeptide CSF114(Glc).

The SPR method presented similar overall results in terms of sensitivity (36%) and specificity (95%) to those previously reported for the ELISA assay, showing that this assay discriminates a group of positive patients (responders) representing a significant sub-population of MS patients [39]. Indeed, MS is a complex disease and four different patients' groups have been defined; anti-glycopeptide antibodies have been shown to be a valuable tool for patient stratification and therapeutic follow-up [40]. A major disadvantage of the SPR methods is in its inherent inability to distinguish IgM *vs.* IgG response, an important element of the response in MS.

Autoantibody detection observed by the complementary immunoassays, even with discordances, could point both SPR and ELISA as useful candidates in diagnosis/prognosis of autoimmune diseases. The presented SPR-based biosensor could be useful in patient diagnosis and accurate monitoring of patient serum antibody levels in response to treatments.

Generally speaking, major development in the field of autoantibodies marked the last 2–5 years. The detection of autoantibodies in an asymptomatic person may have an unexpected importance. Autoantibody may be not only pathogenic or predictive, but some could also be protective, a finding usually quite difficult to challenge. It is anyhow clear, that new methodologies are changing the ways the tests may perform, starting with proposing new antibody/clinic correlation. These approaches have already highlighted the fact that in some autoimmune conditions patients produce more than one autoantibody, with parallel antibody profiles. Different methodologies and multiple autoantibody determination systems should prospectively be used for preventive screening of large populations or at-risk groups, with practical implications of new attempts at earlier diagnosis or the identification of individual patients' autoantibody signatures able to drive the development and selection of different therapies. Owing to major technical differences, not all the assays can offer comparable results raising the need for guidelines for their correct use and interpretation and harmonization on one side, while, from another view, only clinical standardization should assess the value of each testing in a strict clinical, evidence-based, follow up of the results. Here we show that Biacore testing does not fully compare to ELISA and each test results should be independently evaluated. When the clinical role of autoantibodies as disease markers is acknowledged and thus recognized as a good bystander effect of the process, these differences should be extensively studied and clinically validated.

## Figures and Tables

**Table 1.** Summary of all serological results obtained both in SPR and ELISA.

	**Entry**	**Number of samples**	**SPR**	**IgG ELISA**	**IgM ELISA**
**MS patients**	1	2	+	+	+
	2	7	+	+	-
	3	2	+	-	+
	4	9	+	-	-
	5	2	-	+	+
	6	4	-	+	-
	7	4	-	-	+
	8	31	-	-	-

**BD**	9	0	+	+	+
	10	1	+	+	-
	11	1	+	-	+
	12	1	+	-	-
	13	1	-	+	+
	14	2	-	+	-
	15	2	-	-	+
	16	52	-	-	-
